# Denervation induces mitochondrial decline and exacerbates lysosome dysfunction in middle-aged mice

**DOI:** 10.18632/aging.204365

**Published:** 2022-11-04

**Authors:** Matthew Triolo, Debasmita Bhattacharya, David A. Hood

**Affiliations:** 1 Muscle Health Research Centre, York University, Toronto, Ontario M3J 1P3, Canada; 2 School of Kinesiology and Health Science, York University, Toronto, Ontario M3J 1P3, Canada

**Keywords:** mitochondrial biogenesis, autophagy, mitophagy, lysosomes, muscle

## Abstract

With age, skeletal muscle undergoes a progressive decline in size and quality. Imbalanced mitochondrial turnover and the resultant dysfunction contribute to these phenotypic alterations. Motor neuron denervation (Den) is a contributor to the etiology of muscle atrophy associated with age. Further, aged muscle exhibits reduced plasticity to both enhanced and suppressed contractile activity. It remains unclear when the onset of this blunted response occurs, and how middle-aged muscle adapts to denervation. The purpose of this study was to compare mitochondrial turnover pathways in young (Y, ~5months) and middle-aged (MA, ~15months) mice, and determine the influence of Den. Transgenic mt-Keima mice were subjected to 1,3 or 7 days of Den. Muscle mass, mitochondrial content, and PGC-1α protein were not different between Y and MA mice. However, indications of enhanced mitochondrial fission and mitophagy were evident in MA muscle which were supported by a greater abundance of lysosome proteins. Den resulted in muscle atrophy and reductions in mitochondrial protein content by 7-days. These changes occurred concomitant with modest decreases in PGC-1α protein, but without further elevations in mitophagy. Although both autophagosomal and lysosomal proteins were elevated, evidence of lysosome dysfunction was present following Den in MA mice. These data suggest that increases in fission drive an acceleration of mitophagy in muscle of MA mice to preserve mitochondrial quality. Den exacerbates the aging phenotype by reducing biogenesis in the absence of a change in mitophagy, perhaps limited by lysosomal capacity, leading to an accumulation of dysfunctional mitochondria with an age-related loss of neuromuscular innervation.

## INTRODUCTION

Skeletal muscle, which comprises of ~40% of total body mass, is an extremely malleable tissue that undergoes adaptations to changes in contractile activity. As such, the loss of mass with muscle inactivity (i.e., atrophy) is largely due to an upregulation of catabolic processes within myofibers [1, 2]. This can occur in a variety of conditions throughout a lifespan, including prolonged periods of inactivity due to injury and in response to various illnesses [2, 3]. Importantly, the progressive loss of muscle mass and function is a hallmark characteristic of the natural aging process [4], and these age-related deficits have the potential to influence quality of life and lifespan [5]. Although the molecular mechanisms responsible for these changes are not fully characterized, various studies, as reviewed in [6–9] point towards the contribution of mitochondrial homeostasis in both atrophy with chronic disuse models, as well as age-related sarcopenia.

Mitochondria are dynamic organelles that undergo consistent remodeling within skeletal muscle. The process begins with mitochondrial biogenesis, the coordinated expression of nuclear genes encoding mitochondrial proteins (NuGEMPs) with genes from the mitochondrial genome. When portions of the mitochondrial pool become dysfunctional, these segments undergo fission, followed by mitophagy, which is the selective degradation of these organelles through the autophagy-lysosome system. An imbalance in these processes favoring biogenesis could enhance the metabolic capacity of the tissue. However, proper removal is necessary to ensure a well-functioning system; as dysfunctional mitochondria are predisposed to creating more reactive oxygen species (ROS) which can perpetuate damage within the muscle and promote atrophy, as seen in both disuse and aging models [6, 9–11]. Since lysosomes are responsible for the final degradation of mitochondria undergoing mitophagy, they are also implicated in the maintenance of organellar quality throughout a lifespan [12]. Previous work has indicated that muscle from aged rodents possesses deficits in biogenesis with elevations in mitophagy leading to an overall reduction in mitochondrial content [6]. However, the presence of higher mitochondrial ROS in aged muscle suggests that mitophagy may be insufficient to aid in the complete maintenance of organelle health.

Reports from our laboratory as well as others have shown that the skeletal muscle from aged rodents have a blunted adaptive response to stimuli such as chronic contractile activity [13–16], hindlimb re-loading [17], and denervation [18]. The mechanisms responsible for this blunted response remain poorly understood. As neuromuscular denervation is evident in the natural aging process [4, 19, 20] it is of utmost importance to determine how denervation events influences older muscle. Specifically, if the susceptibility to denervation-atrophy in aged muscle is different from that of young muscle, the ramifications on the treatment on age-related sarcopenia must be tailored accordingly. Furthermore, earlier studies have typically compared the muscle from young and aged animals, without consideration for alterations in tissue plasticity in earlier stages of aging (i.e., early aging).

The primary goal of this study is to characterize the time-dependent changes in denervated skeletal muscle from middle-aged mice, with a focus on how mitochondrial turnover is impacted. Utilizing transgenic mt-Keima mice, we were able to assess mitophagy events without pharmacologic intervention. Our results indicate that middle-aged (~15 months) have elevated levels of baseline mitophagy compared to younger animals (~5 months), that is not further increased by short-term, 7-days of denervation. These changes were supported by similar findings in markers of mitochondrial dynamics, mitophagy, and lysosomes at the protein level, which likely serve to maintain the quality of the mitochondrial pool during the early stages of the aging process. However, denervation can accelerate mitochondrial decay, attenuate autophagy and reduce lysosomal function, likely promoting a more rapid deterioration in muscle health in older animals.

## MATERIALS AND METHODS

### Animals

Young (4-6 months) and middle-aged (14-16 months) male mt-Keima mice were utilized in this study. These mice were originally obtained with permission from Drs. Toren Finkel and Nuo Sun (NIH and Ohio State University), and subsequently bred in-house according to standard procedure. Briefly, these mice overexpress a mitochondrial-targeted probe that fluoresces green when exposed to the pH of the cytosol, and red when exposed to the pH of the lysosomes. Increases in the red: green fluorescence are indicative of mitophagy flux [21, 22], as described in detail below. Food and water were provided *ad libitum*. At the appropriate age, mice underwent a denervation protocol (see below).

### Denervation surgery and tissue removal

Mice underwent surgical procedures to induce denervation (Den) of the hindlimb muscles. Briefly, following isoflurane anesthetization, the left sciatic nerve was exposed and transected, whereas the right served as a sham-operated control. Incisions were ~0.5cm in length. Prior to suturing of the surface muscle, sterile ampicillin was administered to the wound site. Next, the skin was closed with metal clips. Animals were provided amoxicillin in their drinking water (0.3mg/L for up to 7 days) and Meloxicam (2mg/kg Day 1, 1mg/kg Day 2, 0.5mg/kg Day 3) was subcutaneously injected for pain management. Mice were randomly assigned to Den for 1, 3, or 7 days, respectively. At each of these time points, animals were held under anesthesia and tissues were extracted for biochemical analysis. Animals were then euthanized via cardiotomy.

### Assessment of mitophagy via mt-Keima

Following the allocated time of denervation, tissue was excised and imaged using confocal microscopy. Briefly, mice were held under anesthetic (isoflurane), and the TA muscle from the sham-operated or denervated limb was removed and placed in ice cold 1xPBS. Next, the surface of muscle was cut thinly in a longitudinal orientation using sharp, surgical scissors. This ~0.5mm thick portion of muscle was placed on a cover slip, dampened with ice cold 1xPBS. Muscles were imaged within 5 minutes of extraction, at room temperature, in which the fluorophore remains stable [21, 22]. The muscle was then visualized and imaged at 20x magnification using an inverted Nikon Eclipse TE2000-U Fluorescence microscope coupled to a Nikon C2 confocal microscope. The fluorophore was excited via sequential laser scanning at 488nm (green) and 561nm (red). Emission was captured at 500-550nm or 575-625nm for green and red, respectively. Settings for all images and quantifications were consistent throughout the study. The individual green and red channels were exported and quantified on a pixel-by-pixel basis using ImageJ software (NIH). Red pixels were quantified as a fraction of total red and green pixels. Due to overlap of the emission spectra of to the green and red channels, representative images appear yellow, representing mitolysosomes. For each replicate, 8 images were taken at random resulting in 32 data points/group.

### Whole muscle protein extracts

The gastrocnemius muscles from the sham-operated and Den hindlimbs were snap frozen in liquid nitrogen following excision from the animal and stored at -80° C. Protein extracts were made by diluting (10x) a small chunk of muscle (~15-20mg) in Sakamoto buffer (20mM HEPES, 2mM EGTA, 1% Triton X-100, 50% Glycerol, 50 mM ß-Glycerophosphate) containing both phosphatase (Sigma) and protease (Roche) inhibitors. Samples were homogenized using a Qiagen TissueLyser II with steel beads and then centrifuged for 15 min at 14,000g. The supernatant fraction was collected and stored at -80° C until further analysis.

### Western blotting

All protein concentrations were determined using the Bradford method. Equal amounts of protein (~25μg) were loaded and separated via SDS-PAGE and transferred onto nitrocellulose membranes (Bio-Rad, Mississauga, ON, Canada). Protein was first visualized with Ponceau stain and then blocked in wash buffer (0.12% Tris-HCl, 0.585% NaCl, 0.1% Tween, pH 7.5) supplemented with 5% skim milk (w/v) at room temperature for 1 hour with gentle agitation. Membranes were then incubated with primary antibodies overnight at 4° C for OXPHOS Cocktail (Ab110413, Lot 2101000654, Abcam), p62 (Ab56416, Lot GR3285986-1, Abcam), LC3-I/II (4108, Lot 3, Cell Signaling Technologies), Bnip3 (gift from Dr. L.A. Kirshenbaum), Parkin (4211, Lot 7, Cell Signaling Technologies), Lamp1 (Ab24170, Lot GR3235632-1, Abcam), V-ATPase B1/2 (sc-55544 F-6, Lot I1018, Santa Cruz), Cathepsin B (D1C7Y, Lot 1, Cell Signaling Technologies), GAPDH (ab8254, Lot GR3317834-1, Abcam). The following day, membranes were washed 3x5minutes in wash buffer and incubated for 1 hour at room temperature with the appropriate HRP-conjugated secondary antibody and subsequently washed 3x5 minutes in wash buffer. The protein density was visualized using enhanced chemiluminescence (1705061, Bio-Rad) with an iBright FL1500 Imaging Station (Fischer Scientific, Oakville, ON, Canada). Band densities were quantified by ImageJ software (NIH) and normalized to corresponding Ponceau stain.

### Statistical analysis

Data were analyzed using GraphPad Prism Software (Version 9) and values are represented as means ± SEM. Student’s unpaired t-tests were utilized to analyze young versus middle-aged control data. A two-way ANOVA was used to assess the interaction between age, denervation and mitochondrial content, whereby significance was achieved at p<0.05. One-way ANOVAs were run to assess the impact of time of Den in the aging cohort; # represents significance p<0.05. Fischer LSD post-hoc test was utilized to assess individual differences in protein levels with Den from age-matched control; * represents a significant difference at p<0.05. p values are shown for trends in the data.

## RESULTS

### Denervation leads to deficits in muscle mass of middle-aged mice at 7 days post-denervation

**Figure 1:**
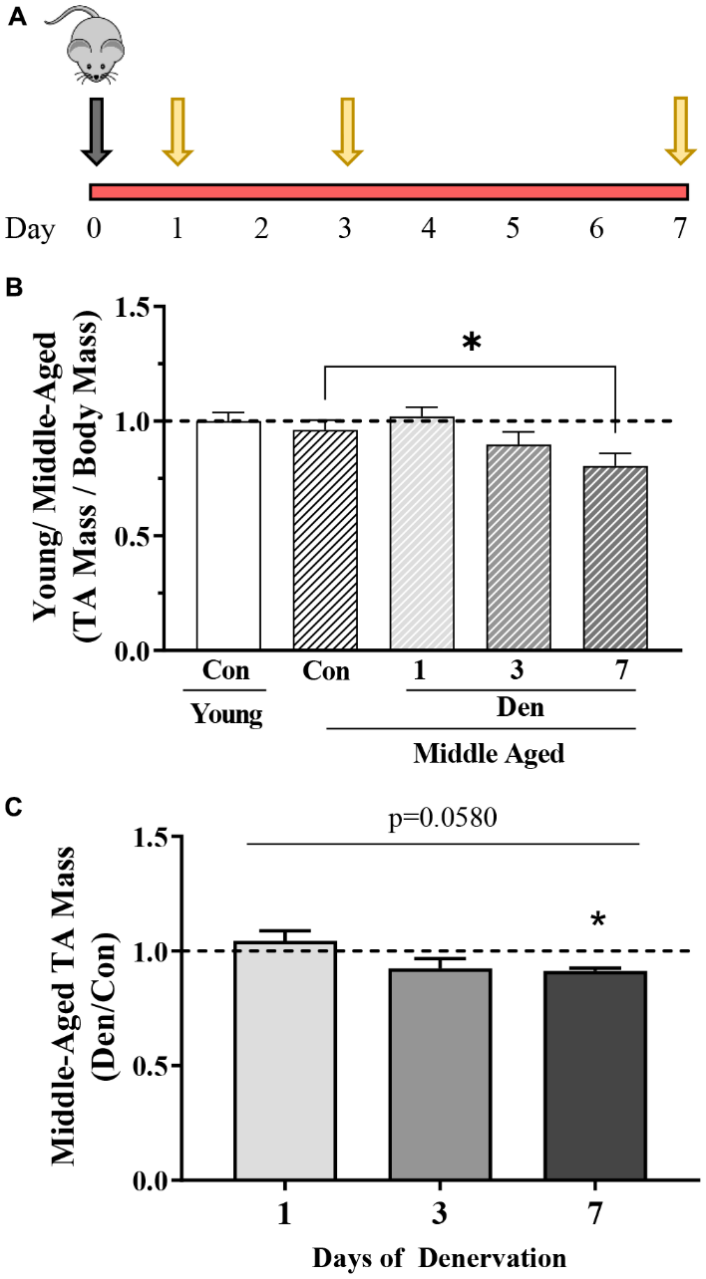
**Overview of study design and changes in muscle mass with age in the absence and presence of 1, 3 or 7 days of denervation.** (**A**) Schematic representation of the study design. Middle-aged mice (MA, ~15 months old) underwent unilateral sciatic denervation for 1, 3 or 7 days. Young mice (YC, ~5 months old) were used as an early adult control. (**B**) Tibialis anterior (TA) muscle mass corrected for body mass in young control, and middle-aged control and 1-, 3- or 7-day denervated limbs. (**C**) TA mass represented as fold of age-matched control. Values are means ± SEM. Main effects of den are represented on graph. *p< 0.05, post-hoc significance. N=12/young control and middle-aged control, N=4/denervation time point.

The experimental design of the study is indicated in Figure 1A. Middle-aged (~15 month) mice were denervated (Den) for 1, 3, or 7 days, whereas the contralateral limb served as a sham-operated control muscle. These middle-aged muscles were compared to control muscle from young, 4–5-month animals. Tibialis anterior (TA) mass was not different in young versus middle-aged mice at this age (Figure 1B). With denervation there was a time-dependent 9% decrease in muscle mass (one-way ANOVA; Figure 1B, 1C) with Den.

### Denervation reduces muscle mitochondrial content in middle-aged mice

**Figure 2:**
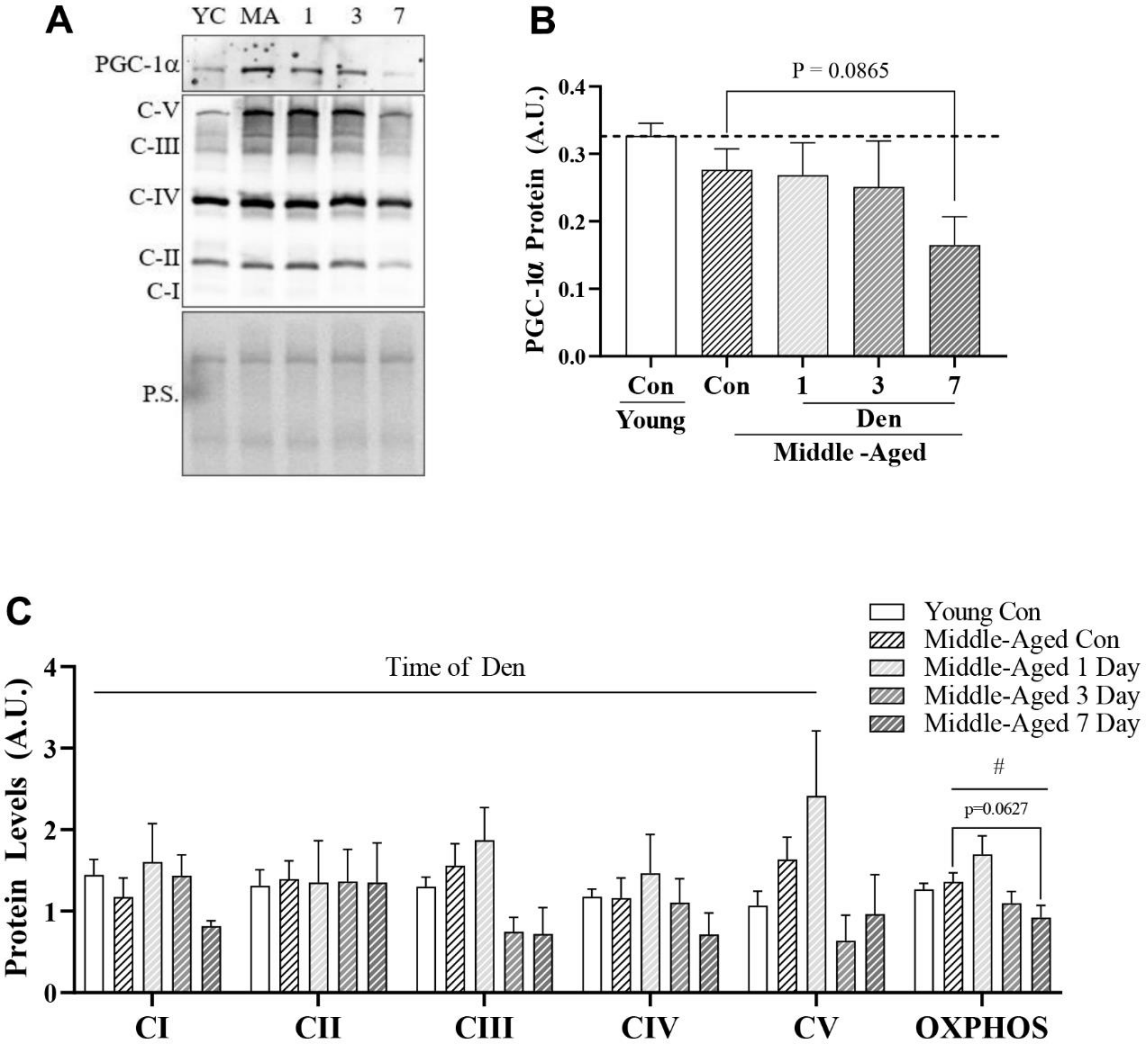
**PCG-1α and mitochondrial protein content in young and middle-aged control and denervated skeletal muscle.** (**A**) Representative western blot for PGC-1α and OXPHOS components. Quantification of (**B**) PGC-1α protein; and (**C**) OXPHOS components and total OXPHOS protein in skeletal muscle. All values were corrected to Ponceau stain (P.S.), and values are reported as means ± SEM, in A.U. Main effects of 2-way ANOVA are represented at p<0.05. # represents significance of a 1-way ANOVA on total OXPHOS protein. Post-hoc trends are represented on graphs. N=12/young and middle-aged control, N=4/denervation time point.

To evaluate mitochondrial content with denervation in middle-aged mice we first measured the protein content of PGC-1α (Figure 2A, 2B). No differences were observed between young and middle-aged muscle, or at 1- and 3-days post-Den. However, we did observe a trending (post-hoc; p=0.0865) decrease of 41% at 7-days post-Den in comparison to age-matched controls. Similarly, mitochondrial content, as indicated by OXPHOS complexes, was not diminished in our middle-aged, compared to our younger cohort. When the effect of time of Den was assessed across all OXPHOS complexes, there was a significant reduction over time (two-way ANOVA, time effect p=0.0116; Figure 2C). We also combined all the OXPHOS protein complex data, excluding CII and uncovered a significant time-effect in the middle-aged mice throughout Den (one-way ANOVA, p=0.0050; Figure 2C). Further analysis revealed a trending 39% decline in mitochondrial protein content at 7-days of den (post-hoc, p=0.0627; Figure 2C).

### Autophagy is upregulated with both aging and denervation

**Figure 3:**
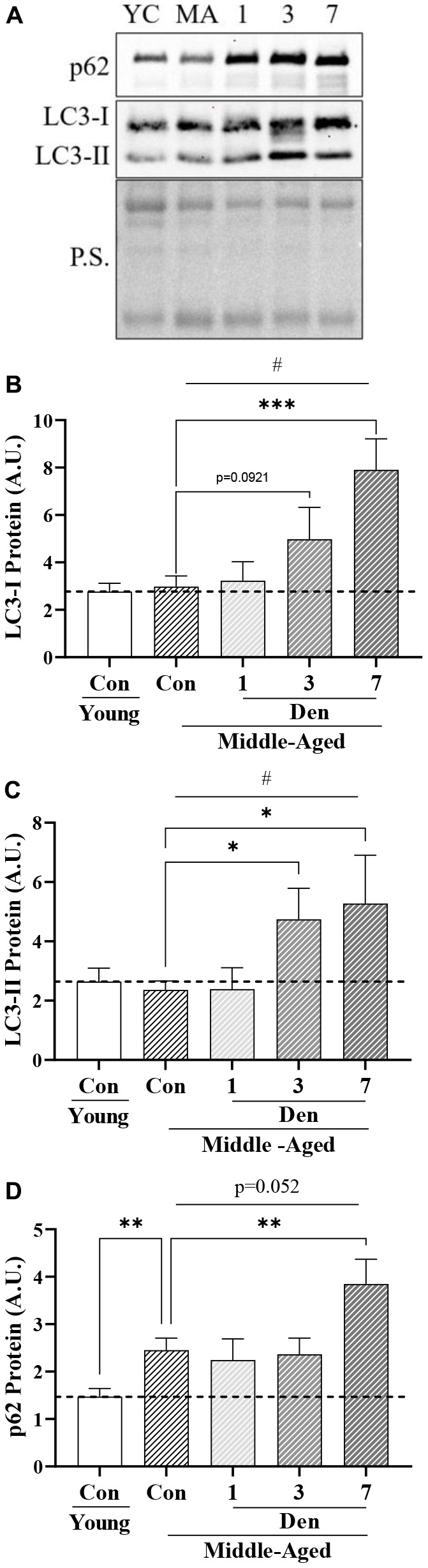
**Autophagosomal proteins in young and middle-aged control and denervated skeletal muscle.** (**A**) Representative western blots for LC3-I, II and p62 protein. Quantification of (**B**) LC3-I protein; (**C**) LC3-II protein; and (**D**) p62 protein in skeletal muscle. All values were corrected to Ponceau stain (P.S.), and values are reported as means ± SEM, in A.U. *p<0.05, t-test between young and middle-aged control. # represents significance of a 1-way ANOVA on protein levels over the course of denervation in middle-aged mice. *p<0.05, **p<0.01, ***p<0.001 represent post-hoc significance. Trends are represented on graphs. N=12/young and middle-aged control, N=4/denervation time point.

Since changes in global autophagy have implications for mitochondrial degradation through mitophagy, we next sought to determine whether aging alone and throughout Den alters autophagosomal protein levels (Figure 3). We uncovered that LC3-I was unchanged in the muscle of middle-aged mice but underwent a significant time-dependent increase throughout the time course of Den (one-way ANOVA, p=0.0023, Figure 3B), manifesting in a significant 2.7-fold elevation at 7 days (post-hoc; p=0.0003, Figure 3B). We also found that autophagosomal LC3-II underwent a time-dependent increase (one-way ANOVA; p=0.0224, Figure 3C). Post-hoc analysis revealed significant 2.0-fold and 2.2-fold greater LC3-II protein from sham-operated control at 3 (post-hoc; p=0.0311, Figure 3C) and 7 days (post-hoc; p=0.0101, Figure 3C) post-Den, respectively. Neither LC3-I or II were changed as a product of age alone, and the LC3-II/I ratio was no different in any group (data not shown). Next, we probed for the autophagosomal-cargo adaptor protein, p62, and found that the muscle from middle-aged animals contained 1.7-fold more protein (t-test; p=0.0042, Figure 3D). p62 protein was further increased by 1.6-fold of aged-matched control at 7 days post-Den (post-hoc; p=0.0129, Figure 3D). These data suggest a modest impairment in autophagy flux with age, that is further exacerbated with denervation.

### Muscle from middle-aged mice displays indices of mitochondrial fragmentation and elevated mitophagy that are unchanged with denervation

**Figure 4:**
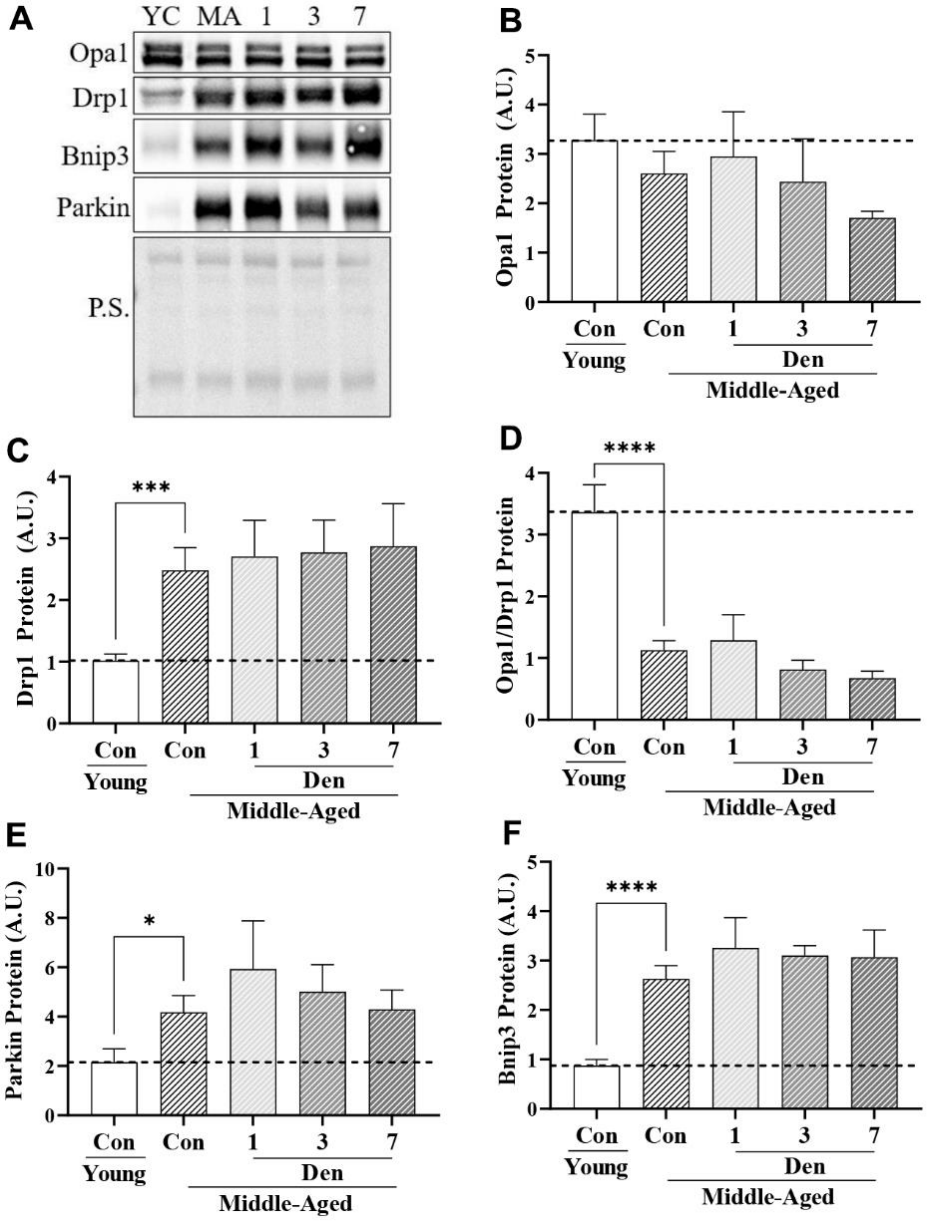
**Proteins related to mitochondrial dynamics and mitophagy in young and middle-aged control and denervated skeletal muscle.** (**A**) Representative western blots for Opa1, Drp1, Bnip3 and Parkin proteins in skeletal muscle samples. Quantification of (**B**) Opa1 protein; (**C**) Drp1 protein; (**D**) Opa1/Drp1 protein; (**E**) Parkin protein; and (**F**) Bnip3 protein. *p<0.05, **p<0.01, *** p<0.001 t-test between young and middle-aged control. N=12/young and middle-aged control, N=4/denervation time point.

To understand whether these changes in global autophagy impact mitochondrial turnover through mitophagy, we first examined whether there were measurable differences in proteins that regulate mitochondrial dynamics. We found that the mitochondrial fusion-mediator, Opa1, was stable with both age and Den (Figure 4B). Alternatively, the mitochondrial fission-related protein Drp1 was elevated by 2.4-fold in the muscle of middle-aged mice (t-test; p=0.0009, Figure 4C), and unchanged throughout the course of Den. These age-related changes were denoted by a 66% decrease in the Opa/Drp1 protein ratio (t-test; p<0.0001, Figure 4D), indicative of a drive for mitochondrial fragmentation. Similar to Drp1, the mitophagy proteins Parkin (t-test; p=0.0276, Figure 4E) and Bnip3 (t-test; p<0.0001, Figure 4F) were upregulated by 1.9- and 3.0-fold, respectively, but remained unchanged with Den.

**Figure 5:**
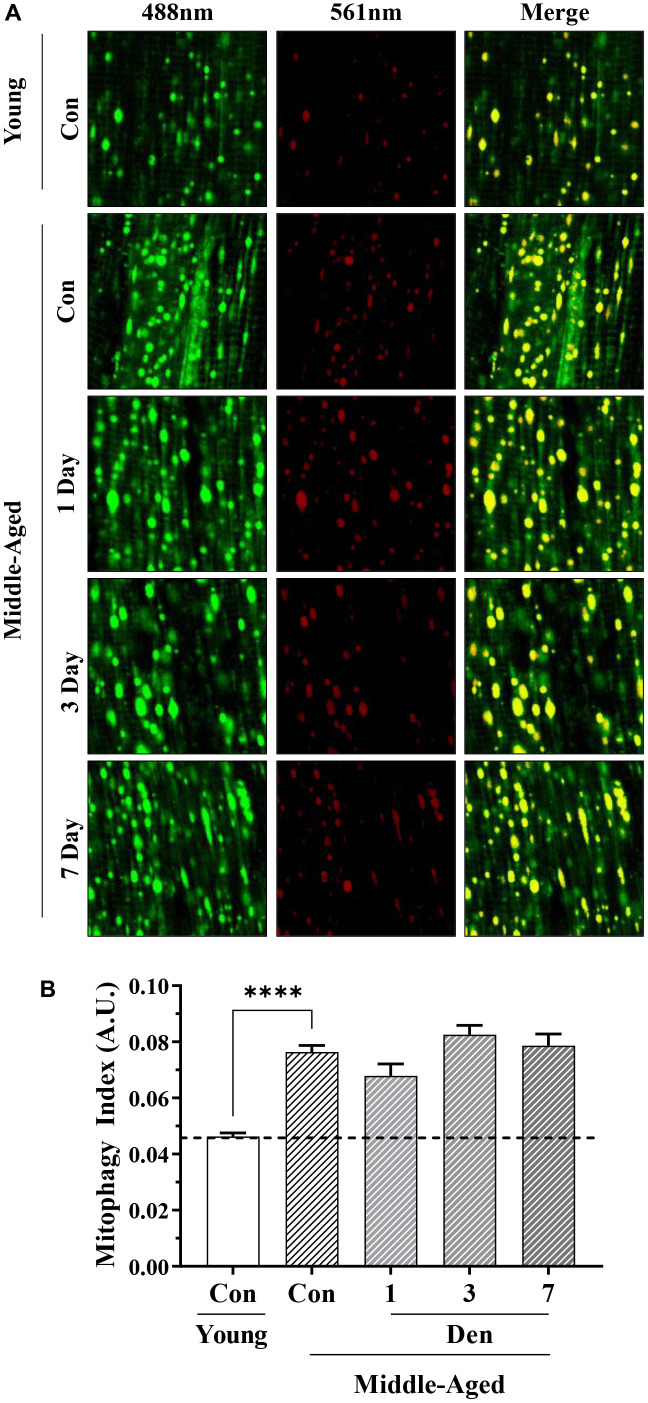
**Assessment of mitophagy flux by mt-Keima fluorescence in young and middle-aged control and denervated skeletal muscle.** (**A**) Representative fluorescence images from the TA muscle of mt-Keima in green (ex. 488nm; mitochondria), red (ex. 561nm; mitolysosomes), and merged. (**B**) Mitophagy index as calculated by red/total red+green fluorescence. ****p<0.0001, t-test between young and middle-aged control. 12 biological replicates were used for young and middle-aged control, 4 biological replicates were used for each denervation time-points. 8 images were taken per biological replicate (N=96/young and middle-aged control, N=32/denervation time point).

We utilized transgenic mt-Keima mice to estimate the degree of change in mitophagy flux with age and denervation. To ensure specificity of this pH-sensitive fluorophore in skeletal muscle we compared the green fluorescence (excitation 488nm) to that of FVB mice (Supplementary Figure 1A). In this assay, we found that the muscle from FVB mice did not fluoresce when excited at the same, or greater, laser power 488nm than that of mt-Keima mice. Furthermore, to determine if the excitation at 561nm is indicative of lysosome-bound mitochondria (i.e., mitolysosomes) as reported previously in other tissue types, a cohort of mice was treated with the microtubule destabilizer, colchicine, for 2-days prior to sampling, which inhibits autophagosomal transport to the lysosomes. We found that colchicine was able to reduce red fluorescence in treated animals, further highlighting the utility of this animal model in skeletal muscle mitophagy assessment (Supplementary Figure 1B). When performing this analysis to compare adult mice to middle-aged mice, we found 65% greater mitophagy events (t-test; p<0.0001, Figure 5A, 5B), indicative of higher basal mitophagy flux with age. Furthermore, we failed to detect any changes throughout the course of Den.

### Lysosomal proteins are upregulated with Den in middle-aged mice, but lysosomal function is reduced

**Figure 6:**
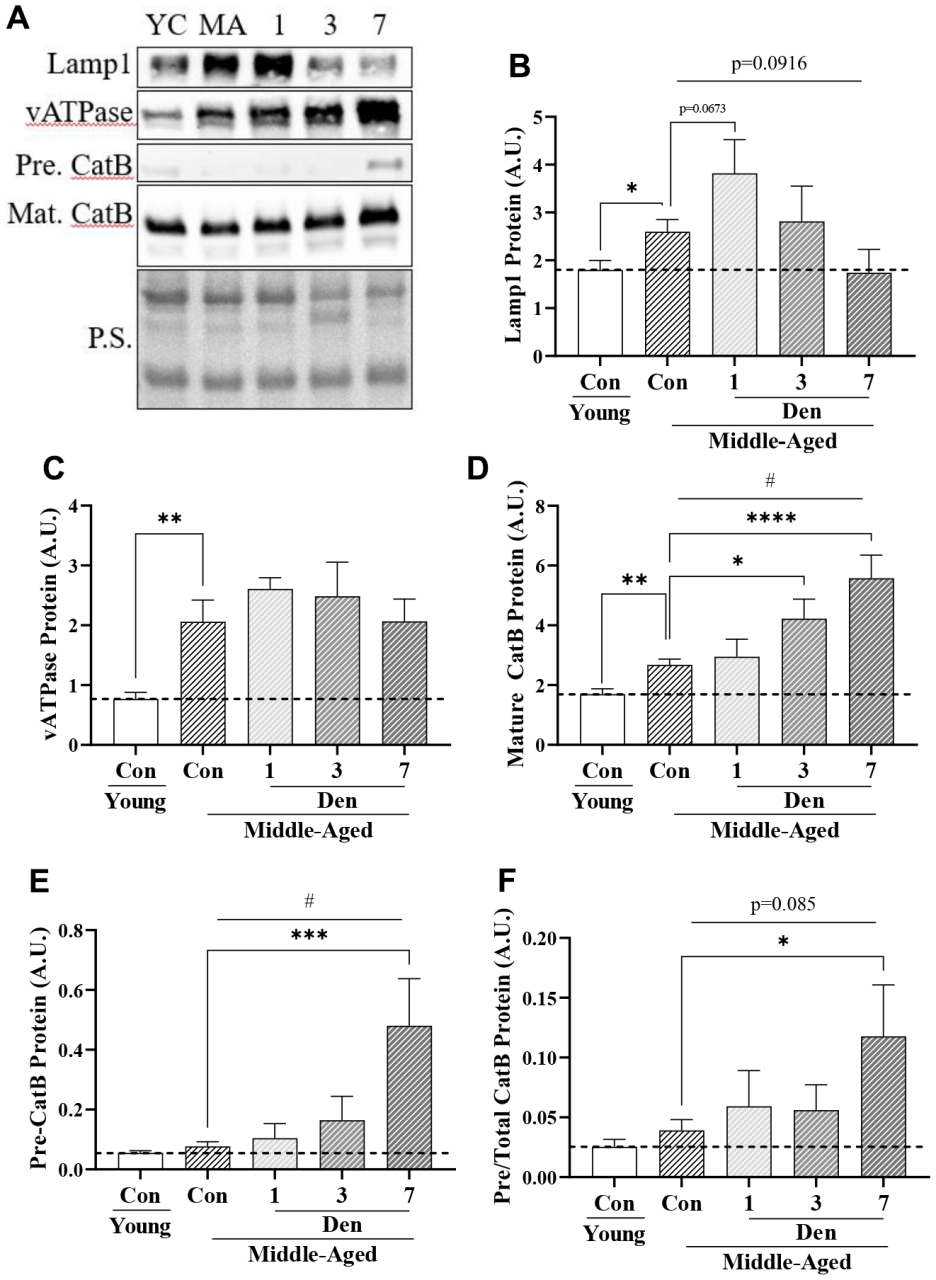
**Lysosome proteins in young and middle-aged control and denervated skeletal muscle.** (**A**) Representative western blots for Lamp1, vATPase, precursor and mature Cathepsin B proteins in skeletal muscle samples. Quantification of (**B**) Lamp1 protein; (**C**) vATPase protein; (**D**) Mature Cathepsin B protein; (**E**) Precursor Cathepsin B protein, (**F**) Precursor/total Cathepsin B protein. All values were corrected to Ponceau stain (P.S.), and values are reported as means ± SEM, in A.U. p<0.05, **p<0.01, t-test between young and middle-aged control. # p<0.05, represents significance of a 1-way ANOVA over the course of denervation. *p<0.05, ***p<0.001, ****p<0.0001, post-hoc significance. N=12/young and middle-aged control, N=4/denervation time point.

To assess the end-stage of the autophagy pathway and to understand why mitophagy flux is unchanged throughout the course of Den in middle-aged adult mice, we evaluated lysosomal protein content (Figure 6). The protein levels of Lamp-1 (t-test; p=0.0233, Figure 6B), vATPase (t-test; p=0.0026, Figure 6C), and mature Cathepsin B (t-test; p=0.0012, Figure 6D) were 1.4-, 2.7- and 1.6-fold greater in the middle-aged cohort, respectively. With Den, Lamp1 protein was further increased at 1-day (post-hoc; p=0.0673, Figure 6B), followed by reductions to control levels at 3- and 7-days. vATPase remained elevated throughout the course of Den. Mature Cathepsin B protein was increased in a time-dependent manner (one way ANOVA; p=0.0006, Figure 6D), resulting in significant 1.6-fold (post-hoc; p=0.0174) and 2.1-fold (post-hoc; p<0.0001) increases at 3- and 7-days post-Den, respectively. The accumulation of immature, precursor, Cathepsin B protein is an index of impaired lysosome formation, and we measured a time-dependent increase in this protein throughout the course of Den (one way ANOVA; p=0.0013, Figure 6E) with a significant increase from age-matched control at 7 days post-Den (post-hoc; p=0.001, Figure 6E). This manifested in a significant 3-fold elevation in pre-Cathepsin B/Total Cathepsin B protein at 7-days post-Den (post-hoc; p=0.0121, Figure 6F), suggestive of impaired lysosome function following Den.

### Mitochondrial decay is better correlated with deficits in biogenesis than changes in mitophagy

**Figure 7:**
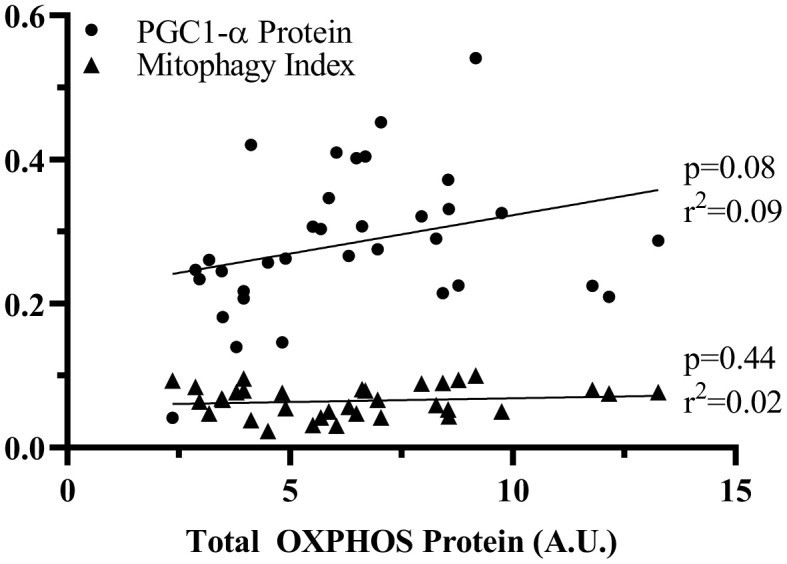
**Correlation of PGC1-α or mitophagy index to OXPHOS protein.** Linear correlations were analyzed to assess the contribution of PGC-1α and mitophagy to change in total OXPHOS protein. p values and r^2^ values are represented on the graph.

To determine whether the decay in mitochondrial content with Den was due to alterations in biogenesis and/or mitophagy, we correlated total OXPHOS protein levels with both PGC-1α, as well as mitophagy index (Figure 7). Mitochondrial content was better correlated with the level of the biogenesis regulator PGC-1α than with mitophagy. This suggests that mitophagy is not regulated by the amount of mitochondrial protein, but more likely related to the quality of organelle pool.

## DISCUSSION

A consequence of aging is the decline in both muscle mass and function, commonly known as sarcopenia [4]. This natural phenomenon, coupled with chronic muscle inactivity accelerates the atrophy of muscle, ultimately hindering one’s quality of life. As there is a rapid increase in the global population of senior adults, there is a growing interest and need to understand the molecular mechanisms of muscle atrophy with combined age and inactivity. Furthermore, there is a lack of data investigating the plasticity of skeletal muscle in early aging (i.e., middle-aged), a time in which early-indices of neuromuscular denervation become apparent [4, 19, 20, 23]. Understanding how motor neuron loss influences skeletal muscle in early aging using a pre-clinical mouse model (i.e., 14-16 month) is vital, as this foreshadows the progress of phenotypic adaptation later on. Thus, in the present study we characterize the plasticity of skeletal muscle in middle-aged animals in response to complete loss of contractile activity via surgical denervation.

The loss of mitochondrial content and function has long been linked to reduced metabolic capacity within muscle. These decrements are evident during both the progression of denervation-atrophy [18, 24] and age-related sarcopenia [6, 10, 25]. Thus, we focused our attention on mitochondrial turnover in young versus aging muscle and examined the adaptability of these organelles to denervation-induced atrophy. Examining mitochondrial turnover *in-vivo* has been challenged by an inability to observe changes in mitophagy without the use of pharmacologic agents such as colchicine. In recent years various fluorescent probes have been designed to examine both bulk autophagy and selective autophagy, including mitophagy, with greater accuracy. Thus, we utilized a novel transgenic mouse model, overexpressing the pH sensitive fluorescent protein, Keima, targeted to the inner mitochondrial membrane via Cox8a presequence [21, 22]. This sensitivity to environmental pH allowed us to simultaneously detect mitochondria contained within the alkaline cytosolic compartment (green fluorescence) and those within the acidic pH of the lysosomes (red fluorescence). The utility of this probe has been shown in various tissue types, including skeletal muscle [21, 24].

In contrast to what is typically observed in older animals [13, 16, 26, 27], we did not observe decrements in mitochondrial protein in our middle-aged cohort in comparison to young counterparts. This absence of organelle loss in the middle-aged muscle was surprising as we measured an increase in indicators of mitophagy flux in the middle-aged cohort. Thus, despite lack of change in PGC-1α protein, other drivers of biogenesis must be elevated to maintain mitochondrial content in the middle-aged cohort. Alternatively, signaling toward mitochondrial biogenesis through PGC-1α may be enhanced irrespective of change in its protein level [28–30]. However, it is likely that this maintained mitochondrial content has an altered morphology compared to younger counterparts in view of the marked changes in proteins regulating organelle fission and fusion that were observed. Specifically, the muscle of middle-aged animals had both greater levels of the mitochondrial fission protein Drp1, and a reduced ratio of Opa1:Drp1 protein. These data suggest a fragmented mitochondrial phenotype in middle-aged muscle, which may precede terminal degradation via mitophagy. As such, it is not surprising that we measured considerable increases in the mitophagy targeting proteins Bnip3 and Parkin, and the mature autophagosome marker, p62. These changes occurred concomitant with a greater abundance of mitolysosomes, as assessed by mt-Keima red fluorescence, and would likely be supported by our measures of more abundant lysosome proteins. Overall, although mitochondrial content was no different in the young muscle from middle-aged mice, there is a clear predisposition for mitochondrial breakdown.

Unlike our previous studies examining time-dependent changes in muscle mass with denervation in young animals [24, 31], the middle-aged mice utilized in the present study show no evidence of muscle atrophy at 3-days post-denervation. Furthermore, although the middle-aged mice utilized in this study show modest decrements in mitochondrial protein content with denervation, these changes are not as robust as those previously measured in young rodents exposed to denervation [24, 31]. Cumulatively, these data indicate a blunted mitochondrial decay and atrophic response to denervation in middle-aged skeletal muscle.

The downregulation of mitochondrial biogenesis has been strongly associated with mitochondrial decay during denervation-induced atrophy in young muscle [32–35]. In the present study, only subtle decrements in PGC-1α protein at 7-days of denervation were observed. These results differ from a past report that uncovered PGC-1α protein decrements at 3-days post neuromuscular denervation [35], suggesting a curtailed deficit in the drive toward mitochondria biogenesis in middle-aged muscle. In our exploration into the contribution of mitochondrial degradation to denervation-atrophy in middle-aged muscle, we found no further increase in the mitochondrial fission signature of these mice, which was maximized by the age-effect. This lack of change was coupled with an inability to upregulate mitophagy proteins in response to denervation, which is typically seen in young muscle [18, 36]. Indeed, no changes in red-fluorescence in our mt-Keima experiments were measured. Importantly, these changes occurred simultaneously with an increase in mature autophagosomal markers, LC3-II and p62. Although no difference in LC3-II/I was observed, a recent analysis by our group correlated these protein markers to flux measures [36], and show that the increase in both these proteins would indicate perturbed global autophagy. This interruption in autophagy may explain the inability to upregulate mitophagy flux in response to denervation within middle-aged skeletal muscle. Cumulatively, these results suggest that decline in mitochondrial biogenesis play a more pronounced role in denervation-atrophy in middle-aged muscle as there is a failure to moderate the mitophagy response. In fact, although not significant, the correlation of PGC-1α to mitochondrial content throughout the course of denervation was a better fit than that of mitophagy. Overall, we believe this reduced plasticity of mitochondrial turnover may influence the adaptability of middle-aged skeletal muscle to denervation.

Autophagy necessitates functional lysosomes to degrade the contents of autophagosomes. Thus, perturbations in lysosome homeostasis have an innate ability to influence mitochondrial degradation and ultimately function of the organelle pool. In fact, muscle from both young rodents exposed to prolonged denervation and sarcopenic animals have been linked to impairments in lysosome health [15, 18, 24]. Normally, lysosomal Cathepsin proteins require an acidic environment for their processing, meaning that the presence of precursor Cathepsin proteins are suggestive of lysosome impairments. Thus, we measured the ratio of Pre-Cathepsin B / total Cathepsin B as an index of lysosome function. We found that the muscle from middle-aged mice contained more lysosome proteins, which appear to retain their functionality at this age, similar to the young cohort. Further, although denervation accelerates the increase in lysosome content, these organelles appear dysfunctional, which may prevent a denervation-induced increase in mitophagy flux. Thus, the increase in lysosome protein with denervation may act as a compensatory mechanism due to a lack of functional capacity to meet the increasing auto- and mitophagic demands within the muscle.

Cumulatively, our data reinforce the concept that as muscle ages, there is an attenuated adaptive plasticity to stimuli placed upon it. We have previously defined this in response to both chronic muscle activity and denervation in rodents that are closer to their maximum lifespan, representing a more dramatic sarcopenic phenotype [15, 18]. In our present study, the inability to upregulate mitophagy flux with denervation is driven by a combination of 1) failure to increase mitophagic proteins and 2) the appearance of dysfunctional lysosomes. Further, the mitochondrial decay observed may be better related to deficits in mitochondrial biogenesis. Importantly, our study is first to show that denervation-induced alterations in mitochondrial turnover and muscle atrophy also occur in middle-aged skeletal muscle, representing a timepoint where sarcopenia and physiologic neuromuscular denervation is not yet apparent. Finally, in the face of aging-induced denervation, the atrophic response may be different than in that of young muscle. Thus, therapies to combat muscle wasting with age-related physiologic denervation must be designed accordingly. Our results imply targeting both mitochondrial biogenesis and maintenance of lysosome capacity will serve to restore mitochondrial homeostasis and likely metabolic capacity of skeletal muscle.

## Supplementary Material

Supplementary Figure 1
