# Supplementary material for: Denervation induces mitochondrial decline and exacerbates lysosome dysfunction in middle-aged mice
Source: Aging (Albany NY). 2022 Nov 4;14(22):8900–13. doi: 10.18632/aging.204365 (PMC9740366; doi:10.18632/aging.204365)
Supplement: Supplementary Figure 1 [file aging-14-204365-s001.pdf]

SUPPLEMENTARY FIGURE

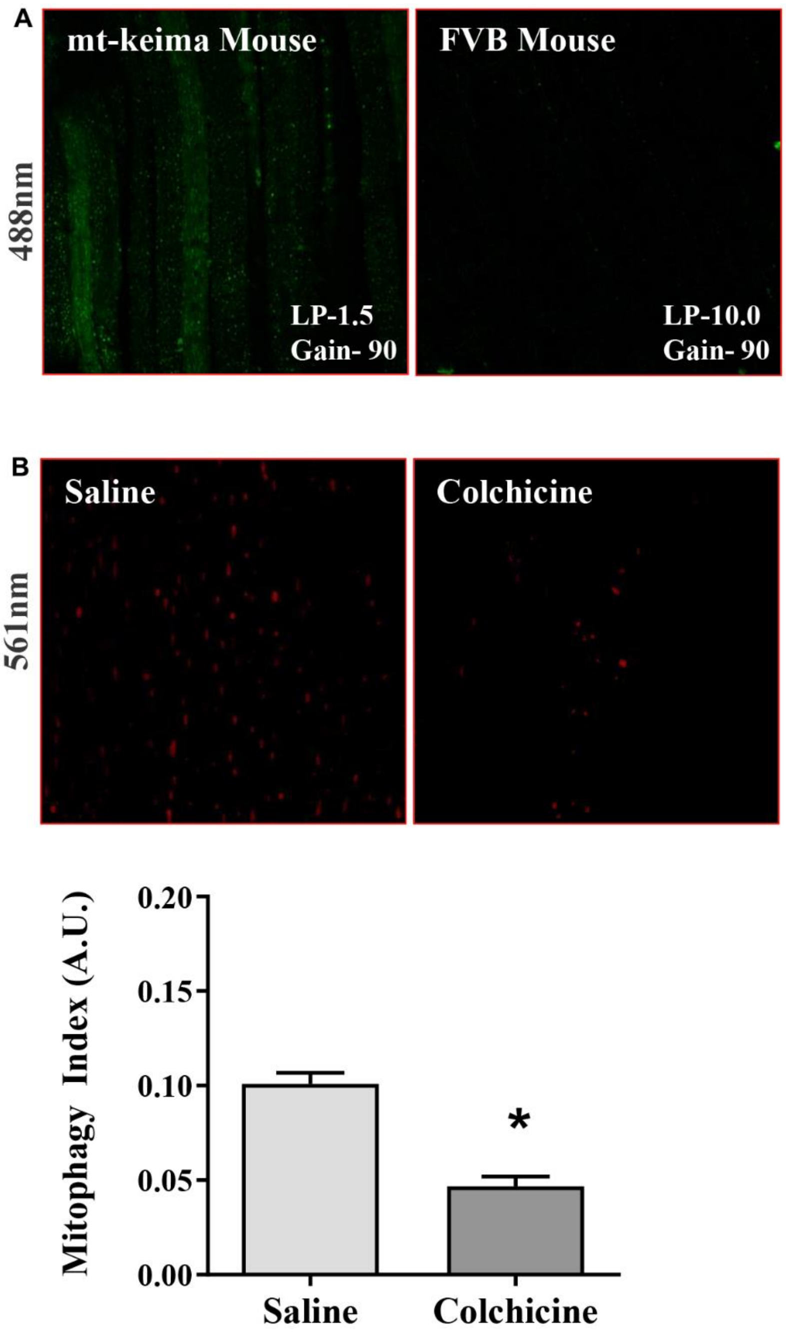

Supplementary Figure 1. Evidence of the utility of mt-Keima mice in detecting mitophagy events within skeletal muscle. (**A**) Wild-type FVB and transgenic mt-Keima mice were imaged for green fluorescence to confirm that the green emission was indicative of mt-Keima. Images are representative confocal microscopy image of the TA muscle excited at 488nm. Laser power was set to a "low" setting to detect keima-green in transgenic mice, whereas laser power was set to a "very high" setting in FVB mice and green fluorescence was not observed. (**B**) mt-Keima mice were treated with saline or 0.4mg/kg/day of colchicine via intraperitoneal injection to show that this microtubule destabilizer reduces red (mitolysosomes) fluorescence in the TA muscle. Representative confocal microscopy images at 561nm and quantifications are shown. All values are mean ± SEM. *represents significance using an unpaired t-test at $p<0.05$.
